# Prevalence of Crimean-Congo haemorrhagic fever in livestock following a confirmed human case in Lyantonde district, Uganda

**DOI:** 10.1186/s13071-022-05588-x

**Published:** 2023-01-07

**Authors:** Stella A. Atim, Marc Niebel, Shirin Ashraf, Patrick Vudriko, Steven Odongo, Stephen Balinandi, Peace Aber, Ronald Bameka, Anna R. Ademun, Charles Masembe, Robert Tweyongyere, Emma C. Thomson

**Affiliations:** 1 College of Veterinary Medicine, Animal Resources and Biosecurity (CoVAB), Makerere University, Kampala, Uganda; 2 Centre of Virus Research (CVR), University of Glasgow, Glasgow, UK; 3 College of Natural Resources (CoNAS), Makerere University, Kampala, Uganda; 4 Department of Emerging, Re-Emerging and Arbovirus Infections, Uganda Virus Research Institute, Entebbe, Uganda; 5 Ministry of Agriculture, Animal Industry and Fisheries, Entebbe, Uganda; 6 Case Western Research Collaboration, Makerere University, Kampala, Uganda; 7 Lyantonde District Local Government, Lyantonde, Uganda; 8 MRC-University of Glasgow Centre for Virus Research, Stoker Building, 464 Bearsden Road, Glasgow, G61 1QH UK

**Keywords:** Crimean-Congo haemorrhagic fever virus, CCHF antibodies, Tick-borne viral infections, CCHF outbreak, Livestock, Animals, Zoonotic disease, CCHF seroprevalence, Uganda

## Abstract

**Background:**

Crimean-Congo haemorrhagic fever (CCHF) is a tick-borne viral infection, characterized by haemorrhagic fever in humans and transient asymptomatic infection in animals. It is an emerging human health threat causing sporadic outbreaks in Uganda. We conducted a detailed outbreak investigation in the animal population following the death from CCHF of a 42-year-old male cattle trader in Lyantonde district, Uganda. This was to ascertain the extent of CCHF virus (CCHFV) circulation among cattle and goats and to identify affected farms and ongoing increased environmental risk for future human infections.

**Methods:**

We collected blood and tick samples from 117 cattle and 93 goats, and tested these for anti-CCHFV antibodies and antigen using an enzyme-linked immunosorbent assay (ELISA), quantitative reverse transcriptase polymerase chain reaction (qRT-PCR) and target enrichment next generation sequencing.

**Results:**

CCHFV-specific IgG antibodies were detected in 110/117 (94.0%) cattle and 83/93 (89.3%) goats. Animal seropositivity was independently associated with female animals (AOR = 9.42, *P* = 0.002), and animals reared under a pastoral animal production system (AOR = 6.02, *P* = 0.019] were more likely to be seropositive than tethered or communally grazed animals. CCHFV was detected by sequencing in *Rhipicephalus appendiculatus* ticks but not in domestic animals.

**Conclusion:**

This investigation demonstrated very high seroprevalence of CCHFV antibodies in both cattle and goats in farms associated with a human case of CCHF in Lyantonde. Therefore, building surveillance programs for CCHF around farms in this area and the Ugandan cattle corridor is indicated, in order to identify opportunities for case prevention and control.

**Graphical Abstract:**

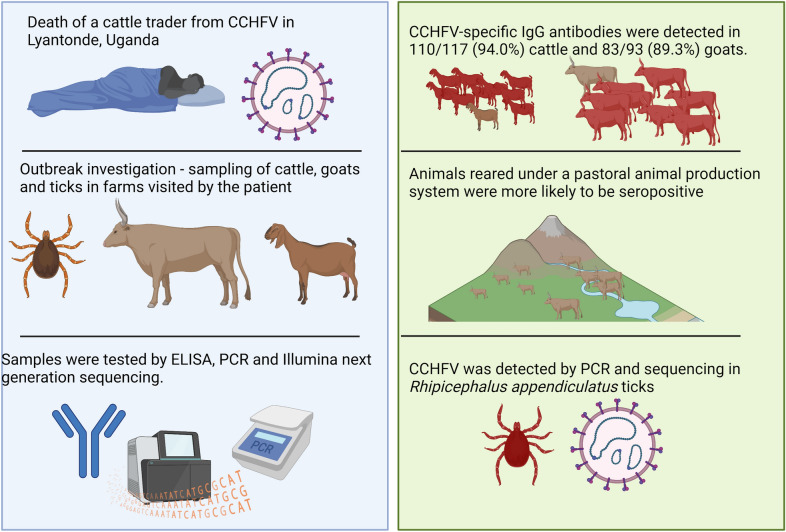

**Supplementary Information:**

The online version contains supplementary material available at 10.1186/s13071-022-05588-x.

## Background

Crimean-Congo haemorrhagic fever (CCHF) is a severe tick-borne zoonotic disease with a high case fatality rate in humans [1]. Overall, the fatality rate has gradually increased during past decades, with important differences across geographical regions and occupations [2]. The aetiological agent has a wide geographical distribution in parts of Africa, Asia, the Middle East and Eastern Europe [3]. Evidence of exposure to CCHF virus (CCHFV) has been reported in non-endemic countries in southern and western Europe [4–6], suggesting that the distribution of endemic countries might further expand within the next years [7].

CCHFV belongs to the genus *Orthonairovirus* and family *Nairoviridae* [8], and displays the typical rapid mutation rate of single-stranded RNA viruses. CCHFV circulates in ticks and is amplified in wild mammalian hosts and livestock [9]. People may become infected with CCHFV from a bite by an infected tick or via blood or fluids of viraemic animals, including humans [1]. Vertebrates, including domestic animals, become infected when bitten by infected ticks. Although domestic animals develop a transient viraemia (7–15 days), they usually remain asymptomatic [7, 10]. Ixodid ticks are infected by vertical transmission or by horizontal transmission when larvae, nymphs or adults take blood meals from viraemic animals or when feeding close to an infected tick (co-feeding infection). The virus persists throughout the tick’s lifespan, leading to repeated opportunities to infect susceptible animals and maintain the virus in the environment [10]

Detection of the virus in animals or in ticks indicates an increased risk of human infection [9]. This study aimed to investigate the seroprevalence of CCHFV exposure in domestic animals following an outbreak involving human cases to ascertain the extent of CCHFV circulation in livestock and to assess the risk for subsequent human infections.

## Methods

### Ethical clearance

This study was undertaken as part of the arboviral infection study (AVI) approved by the Ethics Review Committee at the College of Veterinary Medicine, Animal Resources and Biosecurity of Makerere University, Kampala, Uganda (Reference Number: SVARREC/20/20l8) and by the Uganda National Council for Science and Technology (Reference Number: HS 2485). Informed written consent was obtained from all study participants before they, and their animals, were enrolled in the study.

### Outbreak area

**Fig. 1:**
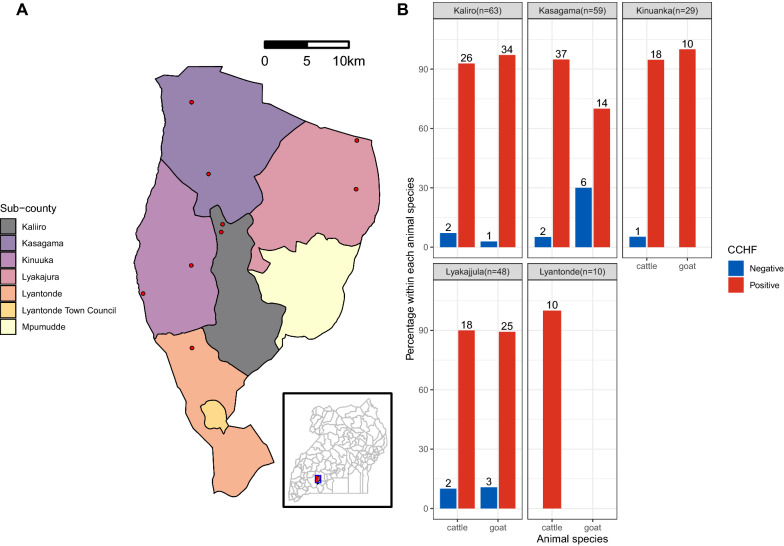
Sample sites of the outbreak study. **a** A map of Lyantonde district showing the sample sites in red and sub-counties as indicated in the legend, and **b** bar charts of seroprevalence by sub-county and animal species. Positive results are shown in red, negative in blue

Lyantonde district is located in the Ankole sub-region in Western Uganda (Fig. 1), bordered by Kiruhura, Sembabule, Rakai and Lwengo districts. Lyantonde occupies 888.1 km^2^, with an estimated population of 93,753 people [11]. Livestock forms the backbone of the economic activity of people in Lyantonde, with the majority (66.1%) rearing cattle and goats of indigenous and exotic crosses under a semi-intensive production system. Kasagama Sub-county (CCHF outbreak site) houses 80% of Lyantonde’s livestock population.

### CCHF case description

On Monday, 29 July 2019, the Kasagama Sub-county One Health team reported the death from suspected viral haemorrhagic fever (VHF) of a 42-year-old male cattle trader, a resident of Kirindimula village, Kisaruwoko Parish Kasagama Sub-county, in Lyantonde district. He had been treated for malaria in Kasagama on 28 July 2019 following a history of fever, headache and vomiting, abdominal pain and general body pains. On Monday, 29 July 2019, he developed haematemesis and epistaxis. He died on Tuesday, 30 July 2019. Samples taken to the Uganda Virus Research Institute (UVRI) Entebbe for analysis and testing on 31 July 2019 indicated that the patient was positive for CCHF and negative for Ebola, Marburg and Rift Valley fever viruses.

### Field investigation

A One Health team comprising the UVRI Ministry of Health, Ministry of Agriculture, Animal Industry and Fisheries, and Lyantonde district local government was convened to undertake the CCHF outbreak investigation in Lyantonde. In order to identify the likely source of the outbreak, an outbreak investigation was carried out on farms purposively selected based on the prior-14-day history of farm visitation by the victim before death from CCHF. A total of 10 farms were identified in this category, and the owners consented to participate in the study. All animals on each farm were placed in a restraint crush and one in four of these were sampled based on random selection and farmers’ choice. For this study, we aimed to collect a total of 100 or more animal samples, based on the numbers used in previous CCHF studies in similar settings in Uganda [12, 13].

A semi-structured herd questionnaire was administered to each farm owner to obtain animal demographic data including age, sex, breed, body temperature and tick infestation number. CCHFV herd level risk factors including herd size, animal production system, tick control practices and history of tick-borne infection were also collected.

Farm animals were selected and blood drawn into sterile EDTA and plain Vacutainer tubes (Becton Dickinson, Plymouth, UK) by veterinary professionals, and transported under cold chain for processing at Mbarara Regional Veterinary Laboratory. Samples were then centrifuged and the serum and plasma aliquoted into 2 ml sterile storage vials (Sarstedt, Inc., Newton, NC, USA). Animal sera were heat-inactivated at 56 °C for 2 h and stored at −80 °C until further laboratory investigation was carried out at the arbovirology laboratory based at UVRI, Entebbe, Uganda.

### Anti-CCHFV immunoglobulin (IgG) detection

CCHFV IgG antibodies were tested in duplicate using the commercial ID Screen^®^ CCHF double-antigen multi-species enzyme-linked immunosorbent assay (ELISA) kit (IDVet Innovative Diagnostics, France) following the manufacturer’s protocol [14]. This kit correlates highly with other assays that are routinely used in CCHF diagnostics [12]. Briefly, the test sera were diluted and incubated at 25 °C for 45 min. The conjugate and substrate steps were all conducted at 25 °C for 30 and 15 min, respectively, before the reaction was stopped. Absorbance was read at 450 nm on an automated ELISA reader (BioTek ELx800, USA) using Gen5 version 2.06 software. The sample positivity percentage (S/P%) for each sample was calculated by dividing the optical density (OD) value of the sample (OD_S_) by the OD of the positive control (OD_PC_), expressed as a percentage. Serum samples were considered positive if the value for their S/P% was greater than 30%.

### CCHFV RNA extraction and quantitative reverse transcriptase polymerase chain reaction (qRT-PCR) testing

Nucleic acid was extracted from plasma using the Beckman Coulter RNA isolation procedure (Brea, CA, USA), following the manufacturer’s instructions. This kit has been found to yield high-quality RNA, suitable for CCHFV nucleic acid testing [15]. Briefly, equal volumes of 200 µl of phosphate-buffered saline (PBS) and test plasma were added to 330 µl lysis buffer and incubated in a water bath at 56 °C for 15 min. After cooling, 410 µl of bind 1/isopropanol solution was added, pipette-mixed and placed on magnetic beads to separate. The supernatant was removed and the beads washed in two subsequent steps using 800 µl of wash buffer/isopropanol and 80% ethanol. This was followed by a DNase treatment step, and the nucleic acid was eluted in 25 µl of nuclease-free water.

The RT-PCR assay was run using the Applied Biosystems 7500 Fast platform and SuperScript III Platinum One-Step qRT-PCR Kit (Invitrogen) according to methods previously described by Atkinson’s assay [16] with slight modifications. The 20 µl reaction volume comprised 10 µL of 2× reaction mix, 1.7 µL of PCR-grade water, 1 µL each of CCHFV reverse and forward primer (at 18 µM working concentration), 0.5 µL of probe (25 µM working concentration) and 5 µL of RNA template. The assay was set to run under the following cycling conditions: 50 °C for 10 min, 95 °C for 2 min, followed by 45 cycles of 95 °C for 10 s and 55 °C for 40 s. A cycle threshold (CT) value greater than 40 was considered negative.

Livestock ticks were collected as described previously [17] from half of the body of domestic animals, while environmental ticks were collected by both dragging and flagging methods. Briefly, ticks were transported in 70% ethanol for identification at the species level using morphological keys [18, 19]. Tick pools were created by collection site, species, sex, and the host animal. All tick pools were then crushed in 0.5 ml of Agencourt lysis buffer in a Geno/Grinder 2000 (OPS Diagnostics, Lebanon, NJ, USA), followed by downstream RNA extraction as described above for plasma (Beckman Coulter).

Tick pools were investigated for the presence of CCHFV, Nairobi sheep disease virus (NSDV) and Dugbe virus (DUGV) genomes using target enrichment next-generation sequencing (NGS), as described previously, using a probe library (Arbocap) targeting all arboviruses including nairoviruses [20]. Viral genomes were detected by de novo assembly using dipSPAdes and IDBA, followed by BLASTn and mapping to relevant nairovirus reference sequences using Tanoti. Maximum likelihood phylogenetic analysis was carried out using IQ-TREE and 1000 ultrafast bootstrap replicates [21].

### Data analysis

Sociodemographic, epidemiological and laboratory data were analysed using Stata software (v15 StataCorp LP, College Station, TX, USA). Demographic and epidemiological characteristics were summarized using frequencies and percentages, stratified by animal species. We estimated the seropositivity of CCHF as the number of samples that tested positive divided by the total tested, expressed as a percentage. We performed both unadjusted and adjusted regression analysis to determine factors associated with CCHFV exposure. For the unadjusted analysis, the association between CCHFV seropositivity in animals and potential risk factors was first assessed using univariate logistic regression analysis. Multicollinearity was examined among different combinations of variables, and where there was a correlation greater than 0.5, we chose a factor most likely to be associated with CCHF exposure. A backward elimination approach was used to remove factors that were not associated with the outcome in the adjusted analysis (*P* > 0.1).

## Results

The demographic characteristics of cattle and goats sampled are presented in Table 1. Briefly, we collected samples from 117 cattle and 93 goats. A total of 89.7% of the cattle were Friesian crossbreeds, aged 2 years or more (73.5%) and predominantly females (95.7%). The average herd size was 124 cattle (standard deviation [SD] = 0.089, 95% confidence intervals [CI] 2.83–3.18). Most cattle had low to moderate tick infestation. A total of 88.2% of the goats sampled were females, 61.3% were Boer crosses, and the average herd size was 63 goats (SD = 0.062, 95% CI 1.76–2.00).

**Table 1:** Sociodemographic and seroprevalence of CCHF in cattle and goats

Characteristics	Cattle (*n* = 117)				Goats (*n* = 93)			
	Total *n* (%)	Negative *n* (%)	Positive *n* (%)	*P*-value	Total *n* (%)	Negative *n* (%)	Positive *n* (%)	*P*-value
Overall seropositivity		7 (6.0)	110 (94.0)			10 (10.7)	83 (89.3)	
Sub-county								
Kaliro	29 (24.8)	2 (6.9)	27 (93.1)	0.949	35 (37.6)	1 (2.9)	34 (97.1)	0.014
Kasagama	39 (33.3)	2 (5.1)	37 (94.9)		20 (21.5)	6 (30.0)	14 (97.1)	
Kinuanka	19 (16.2)	1 (5.3)	18 (94.7)		10 (10.8)	0 (0.0)	10 (100.0)	
Lyakajjula	20 (17.1)	2 (10.0)	18 (90.0)		28 (30.1)	3 (10.2)	25 (89.3)	
Lyantonde	10 (8.6)	0 (0)	10 (100.0)		–	–	–	
Animal sex								
Male	5 (4.3)	2 (33.3)	4 (66.7)	0.180	11 (11.8)	5 (45.5)	6 (54.5)	P < 0.000
Female	112 (95.7)	6 (5.4)	105 (94.6)		82 (88.2)	6 (6.1)	77 (93.9)	
Animal breed								
Indigenous	12 (10.3)	1 (8.3)	11 (91.7)	0.717	36 (38.7)	7 (19.4)	29 (80.6)	0.032
Crossbreed	105 (89.7)	6 (5.7)	99 (94.3)		57 (61.3)	3 (61.3)	54 (94.7)	
Animal age								
< 2 years	31 (26.5)	4 (12.9)	27 (89.1)	0.164	43 (46.2)	6 (14.0)	37 (86.0)	0.289
2–4 years	21 (17.9)	1 (4.8)	20 (95.2)		22 (23.7)	3 (13.6)	19 (86.4)	
> 4 years	65 (55.6)	2 (3.1)	63 (96.9)		28 (30.1)	1 (3.6)	27 (96.4)	
Body temperature								
≤ 38 °C	24 (20.5)	1 (4.2)	23 (93.2)	0.888	41 (44.1)	5 (12.2)	36 (87.8)	0.095
38.1–39.2 °C	74 (63.3)	5(6.8)	69 (93.2)		29 (31.2)	5 (17.2)	24 (82 .8)	
≥ 39.3 °C	19 (16.2)	1 (5.3)	18 (94.7)		23 (24.7)	0 (0.0)	23 (100.0)	
Herd size								
< 50	20 (17.1)	0 (0.0)	20 (100.0)	0.194	21 (22.6)	3 (14.3)	18 (85.7)	0.764
50–99	20 (17.1)	1 (5.0)	19 (95.0)		57 (61.3)	6 (10.5)	51 (89.5)	
100–149	49 (41.9)	2 (4.1)	47 (95.9)		0 (0.0)	0 (0.0)	0 (0.0)	
150–199	9 (7.7)	1 (11.1)	8 (88.9)		15 (16.1)	1 (6.8)	14 (93.3)	
> 200	19 (16.2)	3 (15.8)	16 (84.2)		0 (0.0)	0 (0.0)	0 (0.0)	
Tick count								
No ticks	12 (10.3)	1 (8.3)	11(91.7)	0.540	13 (14.0)	0 (0.0)	13 (100.0)	0.537
< 50	86 (73.5)	6 (7.0)	80 (93.0)		77 (82.8)	10 (13.0)	67 (87.0)	
> 50	19 (16.2)	0 (0.0)	19 (100.0)		3 (3.2)	0 (0.0)	3 (100.0)	
Production system								
Tethering	14 (12.0)	2(14.3)	12 (85.7)	0.168	20 (21.5)	6 (30.0)	14 (70.0)	0.011
Fence/paddocks	39 (33.3)	3 (7.7)	36 (92.3)		38 (40.9)	3 (7.8)	35 (92.1)	
Pastoralism	64 (54.7)	2 (3.1)	62 (96.8)		35 (37.6)	1 (2.9)	34 (97.1)	
Acaricide application								
Once a week	117 (100.0)	7 (6.0)	110 (94.0)	–	93 (100.0)	10 (10.8)	83 (89.2)	–
History of tick-borne diseases								
Anaplasmosis	20 (17.1)	1 (5.0)	19 (95.0)	0.658	15 (100.0)	1 (6.7)	14 (93.3)	–
East Coast fever	97 (82.9)	6 (6.2)	91 (93.0)		–	–	–	

Demographic characteristics and CCHF seropositivity clustered by animal species

Temperature (°C) is rectal temperature of the animals measured in degrees Celsius, *N* is the total number of samples tested for the respective animal species, *n* is CCHF seroprevalence in numbers and % is seroprevalence expressed as percentages for the respective characteristic

CCHFV antibodies were detected in 110 (94.0%) out of 117 cattle and 83 (89.3%) out of 93 goats tested, with no statistical difference in CCHF seropositivity between cattle and goats (*P* = 0.208) (Table 1). For the unadjusted model (Table 2), CCHF seropositivity was significantly associated with animal production system [(fence/paddocks: unadjusted odds ratio [UOR] = 3.64, 95% CI 1.15–11.50, *P* = 0.028), (pastoralism: UOR = 9.85, 95% CI 2.43–39.76, *P* = 0.001)], all compared with tethered or communally grazed animals. Mature animals over 4 years of age (UOR = 4.69, 95% CI 1.24–17.71, *P* = 0.023) and female animals (UOR = 9.93, 95% CI 3.05–32.34, *P* = 0.0001) were also associated with seropositivity.

**Table 2:** Risk factors associated with CCHF exposure in animals

Risk factor	Attribute	Univariate analysis		Multivariate analysis	*P*-value
		Odds ratio (95% CI)	*P*-value	Odds ratio (95% CI)	
Sub-county	Kaliro	Ref		Ref	
	Kasagama	0.31 (0.08–1.24)	0.099	–	–
	Kinuanka	1.38 (0.14–13.83)		–	–
	Lyakajjula	0.42 (0.09–1.86)		–	–
	Lyantonde TC	–		–	–
Livestock production system	Tethering/communal	Ref		Ref	
	Fence/paddock	3.64 (1.15–11.50)	0.028	2.89 (0.81–10.32)	0.102
	Pastoralism	9.85(2.43–39.76)	0.001	6.02 (1.34–27.09)	0.019
Animal species	Goats	Ref		Ref	
	Cattle	1.89 (0.69–5.18)	0.214	–	–
Animal breed	Indigenous	Ref		Ref	
	Crossbreed	1.03 (0.12–8.53)	0.975	–	–
Animal sex	Male	Ref			
	Female	9.93 (3.05–32.34)	< 0.0001	9.42 (2.29–38.71)	0.002
Animal age	< 2 years	Ref		Ref	
	2–4 years	1.52 (0.46–5.19)	0.501	–	–
	> 4 years	4.69 (1.24–17.71)	0.023	–	–
Herd size	< 100	Ref			
	100–200	1.98 (0.48–5.29)	0.444		
	> 200	0.49 (0.12–1.98)	0.321		
Animal body temperature	≤ 38 °C	Ref			
	38.1–39.2 °C	0.95 (0.33–2.74)	0.918	1.20 (0.37–3.86)	0.763
	≥ 39.3 °C	4.17 (0.48–35.94)	0.194	10.82 (0.93–125.82)	0.057
Tick infestation	No tick	Ref			
	< 50 ticks	1.21 (0.13–11.63)	0.866	–	–
	> 50 ticks	0.57 (0.05–6.61)	0.654	–	–

Logistic regression results for risk factors associated with CCHF seropositivity in animals (significant associations at *P* < 0.05)

*Ref* indicates the reference variable for the characteristics listed

Dash (–) denotes variables dropped out of the final model because their probability entries were higher than 0.1

In the multivariable regression model, CCHF seropositivity was independently associated with female animals (adjusted odds ratio (AOR) = 9.42, 95% CI 2.29–38.71, *P* = 0.002) and animals reared under the pastoral production system (AOR = 6.02, 95% CI 1.34–27.09, *P* = 0.019] compared with those tethered or communally grazed.

**Fig. 2:**
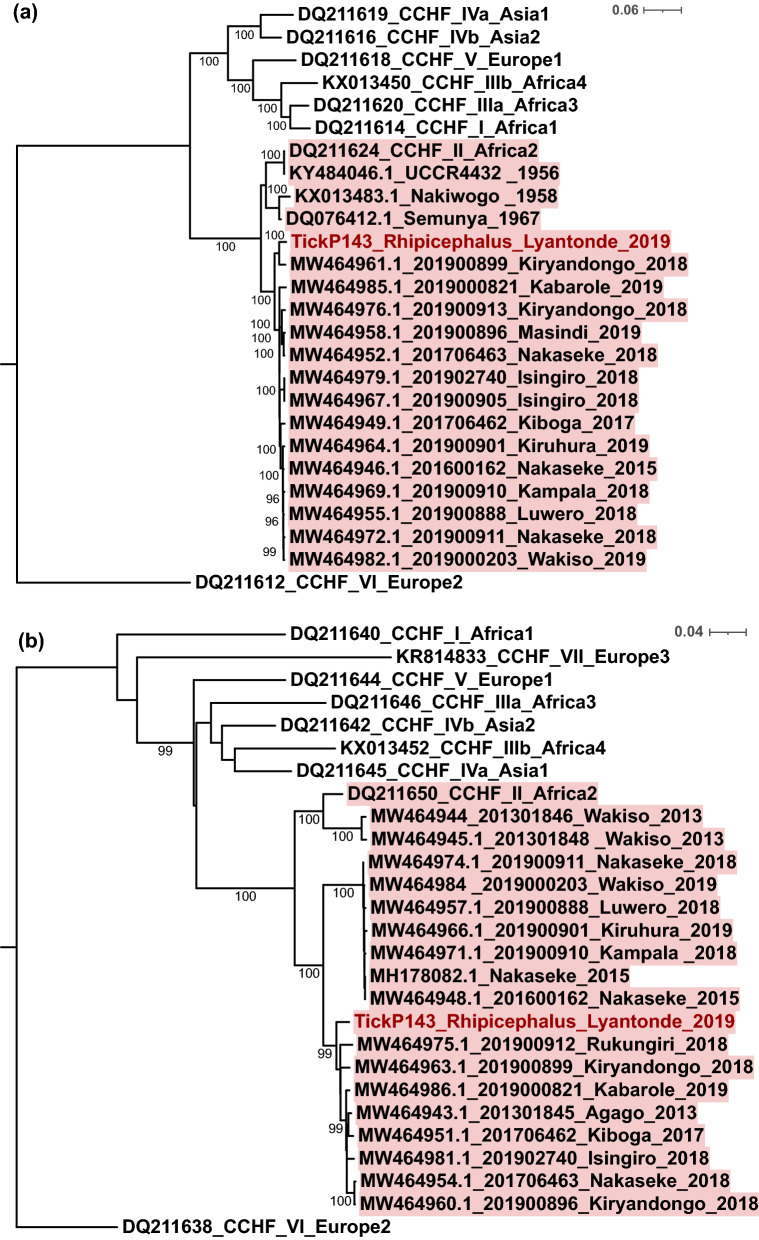
Maximum likelihood tree for CCHFV. **a** L and **b** S segments of Ugandan tick isolate 2019, constructed using IQ-TREE with 1000 ultrafast bootstraps and substitution model GTR+F+I+G4 for nucleotide sequences representative of different lineages. Tree scale indicates substitution events. Sequence sampled in this study is shown in red, the Africa II lineage is highlighted in red; Ugandan sequences show region and year of sampling

All domestic animal samples tested negative for CCHFV on RT-PCR. However, we detected CCHFV in *Rhipicephalus appendiculatus* in ticks collected from cattle on one farm area (Kaliro Sub-county) visited by the affected patient. Phylogenetic analysis is shown in Fig. 2. The TickP143 Lyantonde 2019 sequence clusters closely with isolates derived from human CCHFV infections between 2013 and 2019.

## Discussion

In this outbreak investigation study, we aimed to investigate the source of CCHF infection in a human cattle trader and associated risk factors in the area. Livestock are infected when bitten by infected ticks, and develop asymptomatic transient viraemia lasting 7–15 days. Humans may become infected via contact with the blood or fluids of these viraemic animals or by tick bites. Ticks may remain infected with CCHF throughout their lifespan and act as the main reservoir of CCHF. In keeping with this, we detected CCHF in *R. appendiculatus* ticks as part of the outbreak investigation, as shown in the phylogenetic analysis with our tick-derived sequence clustering with recently sequenced human cases from Uganda. CCHFV RT-PCR did not reveal PCR-positive animals 9 days after the diagnosis of the human case. This may suggest that the individual was infected by a tick bite rather than direct contact with viraemic animals or that affected animals had become aviraemic after an initial viraemic phase. Vertebrates, including domestic animals, when infected with CCHFV develop transient viraemia lasting for about 2 weeks [9]. Trading of livestock was previously implicated in a CCHF outbreak in India and may be more common than previously reported in other parts of the world, including Uganda [22]. In the farms visited by the cattle trader in this study, the overall seropositivity was extremely high in both cattle (94.0%) and goats (89.3%). We used a commercial CCHF ELISA previously optimized to reduce cross-specific results. Nevertheless, we have previously shown this assay to exhibit cross-reactivity with antibodies targeting the related Dugbe and Nairobi sheep disease orthonairoviruses [17]. We have also detected these viruses in Uganda by sequencing of ticks (less frequently than CCHFV) but did not detect them in our study in Lyantonde. The absence of other orthonairoviruses in ticks in Lyantonde suggests that seropositivity is more likely to relate to CCHFV in this area, but further, more extensive studies are indicated. We also noted the detection of other RNA viruses in the domestic animal samples (Additional file 1: Table S1) using methods that we have developed for NGS over a number of years [20, 23, 24].

In Uganda, previous reports have indicated that over 65% of human cases in the country occur among animal handlers [25]. Evidence of CCHFV prevalence in humans and ticks has been documented in this and other studies [26–28]; however, serosurvey of CCHF in livestock either as part of a viral haemorrhagic fever surveillance program or during outbreaks has not been well described. We report a very high CCHFV seroprevalence in both cattle and goats during an ongoing outbreak, with no interspecies statistical differences, in an area associated with a fatal human infection. A higher seroprevalence in cattle (94.0%) was noted compared with other studies previously conducted in Uganda [12] and nearby countries such as Kenya, Sudan, the Democratic Republic of the Congo, Zambia, Malawi and South Africa [29–36]. Similarly, a higher seroprevalence was noted in goats (89.3%) in comparison with results obtained in different regions of Africa, including Mauritania [37], Nigeria [38] and Senegal [39].

The occurrence of CCHFV antibodies in livestock has been shown to vary depending on serological methods used and host factors including age, sex, breed, livestock management system, vector abundance and competence [12, 31, 34]. Similar to livestock serosurvey conducted in Cameroon [40], our independent regression analysis showed an increased risk of CCHFV seropositivity among animals reared under a pastoral production system. Up to 50.9% of the animals surveyed were occasionally moved to neighbouring districts and wildlife-protected areas of Lake Mburo National Park in search of water and pasture. Domestic animals may acquire and transmit ticks within wildlife grazing grounds, with the risk of inter-district spread, thereby increasing the risk of tick-borne diseases in the area. We also found a significant association between female animals and CCHF seropositivity. Additionally, 94% of the animals reared were hybrid animals and would most likely be kept for a longer time because of their high production value; therefore, they may be more exposed to tick bites.

## Conclusion

This investigation demonstrated an extremely high seroprevalence of CCHFV antibodies in both cattle and goats following the occurrence of a fatal human CCHF case in Lyantonde, Uganda. Further research and efforts to improve case prevention and control are indicated, including building surveillance programs for CCHF around farms and the interface between wild hosts, livestock and humans in the cattle corridor in Uganda. Sampling of domestic animals across the country may provide vital information on the ongoing risk to humans in different geographical areas.

## Supplementary Information

**Additional file 1: Table S1. **Viruses detected by NGS following RNA extraction in domestic animal samples.

## Data Availability

All data collected during the outbreak investigation were analysed, and included in the manuscript for publication. Raw data are available on request.
