# Supplementary material for: Prevalence of Crimean-Congo haemorrhagic fever in livestock following a confirmed human case in Lyantonde district, Uganda
Source: Parasit Vectors. 2023 Jan 7;16:7. doi: 10.1186/s13071-022-05588-x (PMC9824997; doi:10.1186/s13071-022-05588-x)
Supplement: Supplementary file 1 — Additional file 1: Table S1. Viruses detected by NGS following RNA extraction in domestic animal samples. [file 13071_2022_5588_MOESM1_ESM.docx]

**References**

**Additional file 1: Table S1. Viruses detected by NGS following RNA extraction in domestic animal samples**

Bovine_fever_ephemerovirus

Bovine_retrovirus_CH15

Enzootic_nasal_tumour_virus_of_goats

Hepacivirus

Jaagsiekte_sheep_retrovirus

Ledantevirus

Moloney_murine_sarcoma_virus

Nyangole_orthobunyavirus

Odocoileus_hemionus_endogenous_retrovirus

Orf_virus

Orungo_virus

Pegivirus

Reticuloendotheliosis_virus

Rous_sarcoma_virus

Simian_retrovirus_Y

Sindbis_virus

Spodoptera_frugiperda_ascovirus_1a

Ungulate_copiparvovirus_1
